# Transient Osteoporosis of Pregnancy Affecting the Ankles: A Case Report and Literature Review

**DOI:** 10.7759/cureus.106478

**Published:** 2026-04-05

**Authors:** Yuki Terashima, Yuji Miyoshi, Yuichi Nagase

**Affiliations:** 1 Department of Rheumatic Diseases, Tokyo Metropolitan Tama Medical Center, Tokyo, JPN; 2 Department of Rheumatic Surgery, Tokyo Metropolitan Tama Medical Center, Tokyo, JPN

**Keywords:** ankle, bone marrow edema, magnetic resonance imaging, pregnancy, radiography, transient osteoporosis of pregnancy

## Abstract

Transient osteoporosis of pregnancy (TOP) is a rare condition that typically presents during the third trimester as unexplained lower limb joint pain, most commonly involving the hip. We report an uncommon case of TOP affecting both ankles and provide a literature review of previously reported cases involving the ankle. A 31-year-old Japanese woman developed bilateral ankle pain in the second trimester of pregnancy without any history of trauma. She presented to the orthopedic outpatient clinic in the postpartum period, and her radiographs revealed bilateral diffuse osteopenia, which brought her to our rheumatologic department. Additional radiographic evaluation of the spine and hips showed no evidence of osteopenia. Magnetic resonance imaging of the right ankle, which was obtained for worsening pain, demonstrated bone marrow edema. Blood tests, which evaluated inflammatory markers and alkaline phosphatase, and a musculoskeletal ultrasound were unremarkable. The patient’s symptoms gradually resolved with conservative management, including vitamin D supplementation, intermittent use of nonsteroidal anti-inflammatory drugs, and rest. At three months postpartum, radiographs showed partial improvement of osteopenia, whereas MRI still demonstrated bone marrow edema. Follow-up radiographs at eight months demonstrated resolution of the patient’s osteopenia, which was consistent with a diagnosis of TOP. We also reviewed previously reported cases of TOP affecting the ankles, identifying eight cases in total. Clinicians should consider the possibility of TOP in pregnant or postpartum women presenting with unexplained joint pain in the lower limbs, including the ankles, even when the pain appears during the second trimester of pregnancy. TOP carries a risk of fracture, making timely and appropriate conservative management essential. Although based on a single case, these findings may indicate that radiographs could reflect recovery earlier than MRI, and may be useful as a follow-up imaging modality in TOP affecting the ankles.

## Introduction

Transient osteoporosis of pregnancy (TOP), first described by Curtiss and Kincaid in 1959 [1], is a rare, self-limiting skeletal condition characterized by unexplained lower limb joint pain and a reduced range of motion without any trigger or trauma. Typically, TOP manifests as hip joint pain during the third trimester of pregnancy [2]. The specific etiology of TOP remains unknown, although it is thought to be related to changes in perinatal bone metabolism and mechanical compression of the sympathetic nerves and veins, leading to intraosseous hypervascularity [3,4]. Approximately 30 g of calcium is transferred to the fetus during pregnancy, 80% of which occurs during the third trimester, possibly triggering TOP in susceptible pregnant women [5]. Although current evidence for the treatment of TOP is limited to findings from case reports [6], standard management is generally conservative and includes rest, temporary non-weight-bearing or reduced weight-bearing to minimize the risk of insufficiency fractures, and analgesics. Calcium and vitamin D supplementation is recommended if a deficiency is present. Discontinuation of breastfeeding is not routinely recommended but may be considered in selected cases, taking into account symptom severity, patient preferences, and shared decision-making, to promote the normalization of postpartum bone metabolism. A previous case report reported the use of bisphosphonates, calcitonin, and core decompression surgery; however, further studies are needed to assess the safety and effectiveness of these treatments [7,8].

TOP is likely underdiagnosed in clinical practice, with an estimated prevalence of one in 250,000 pregnant women in the third trimester [2,9]. Most reported cases involve the hip joint, and complications such as fractures and functional impairment, which may necessitate cesarean section, highlight the importance of considering TOP in patients with persistent joint pain during the perinatal period. TOP affecting the ankles is uncommon, and only a few cases have been reported to date. Here, we present the case of a postpartum woman who developed TOP affecting the ankle during her second trimester and a literature review of previous case reports of TOP affecting the ankle.

## Case presentation

**Figure 1:**
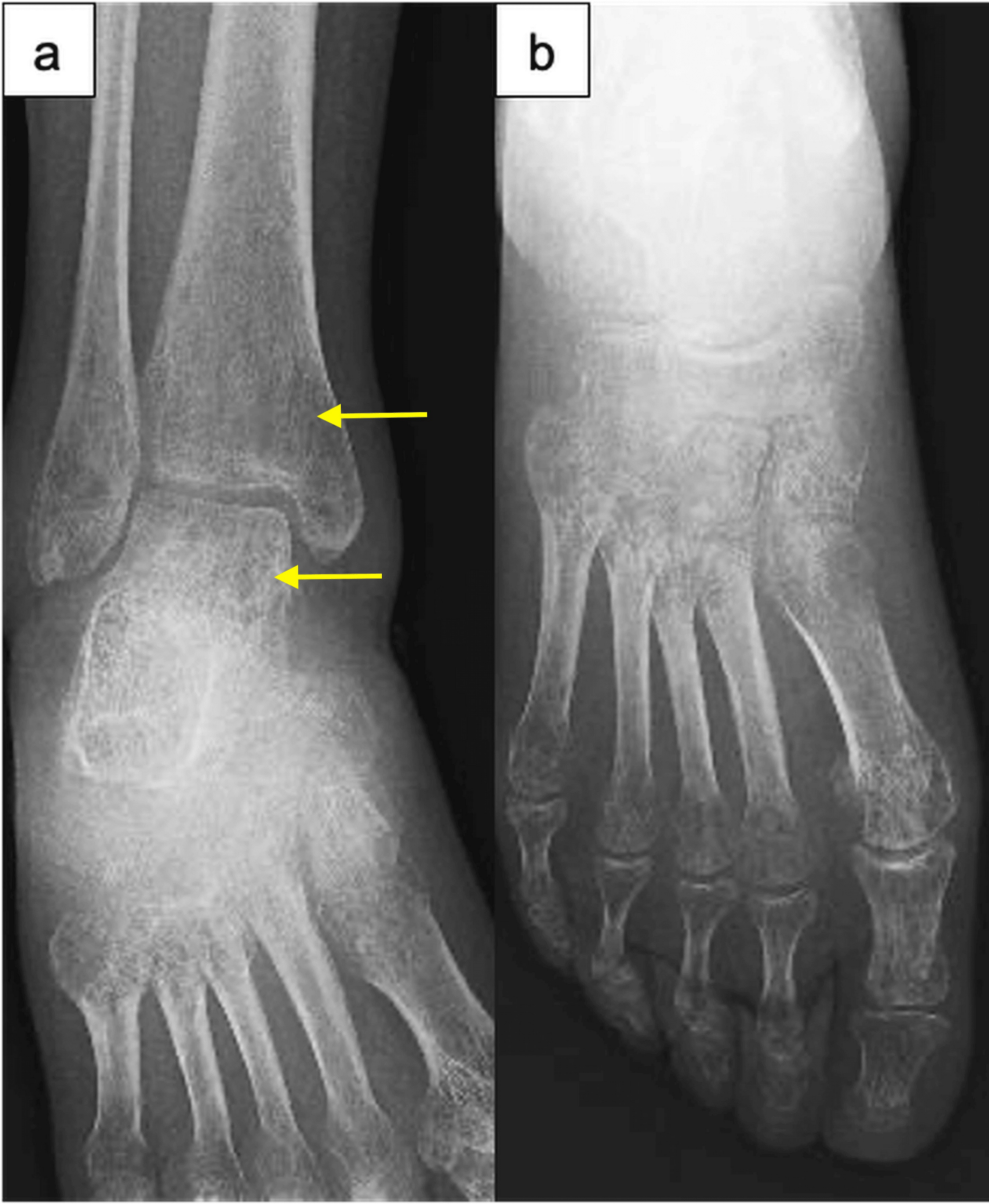
Serial anteroposterior radiographs of the right ankle and foot. At 1 week postpartum: (a) diffuse osteopenia in the distal tibia and fibula; (b) diffuse osteopenia in the calcaneus, navicular, cuboid, cuneiform, and phalanges. Arrows indicate representative areas of osteopenia in the distal tibia and talus.

A 31-year-old primigravida presented to an orthopedic clinic at 24 weeks gestation with right ankle pain. She had no significant comorbidities or regular medication use. Owing to her pregnancy, imaging studies were not performed at the initial clinic, and she was conservatively managed with analgesics. This decision may have been influenced by concerns about fetal radiation exposure. At 36 weeks gestation, her right ankle pain worsened, and she developed new-onset pain in the contralateral ankle. Her ability to perform activities of daily living (ADLs) was impaired due to the ankle pain. She subsequently delivered vaginally at full term. During the first week postpartum, she consulted another orthopedic clinic where bilateral ankle radiographs revealed severe osteopenia affecting the distal ends of the tibia and fibula, talus, navicular, cuboid, cuneiform, calcaneus, and phalanges (Figure 1a, 1b).

**Figure 2:**
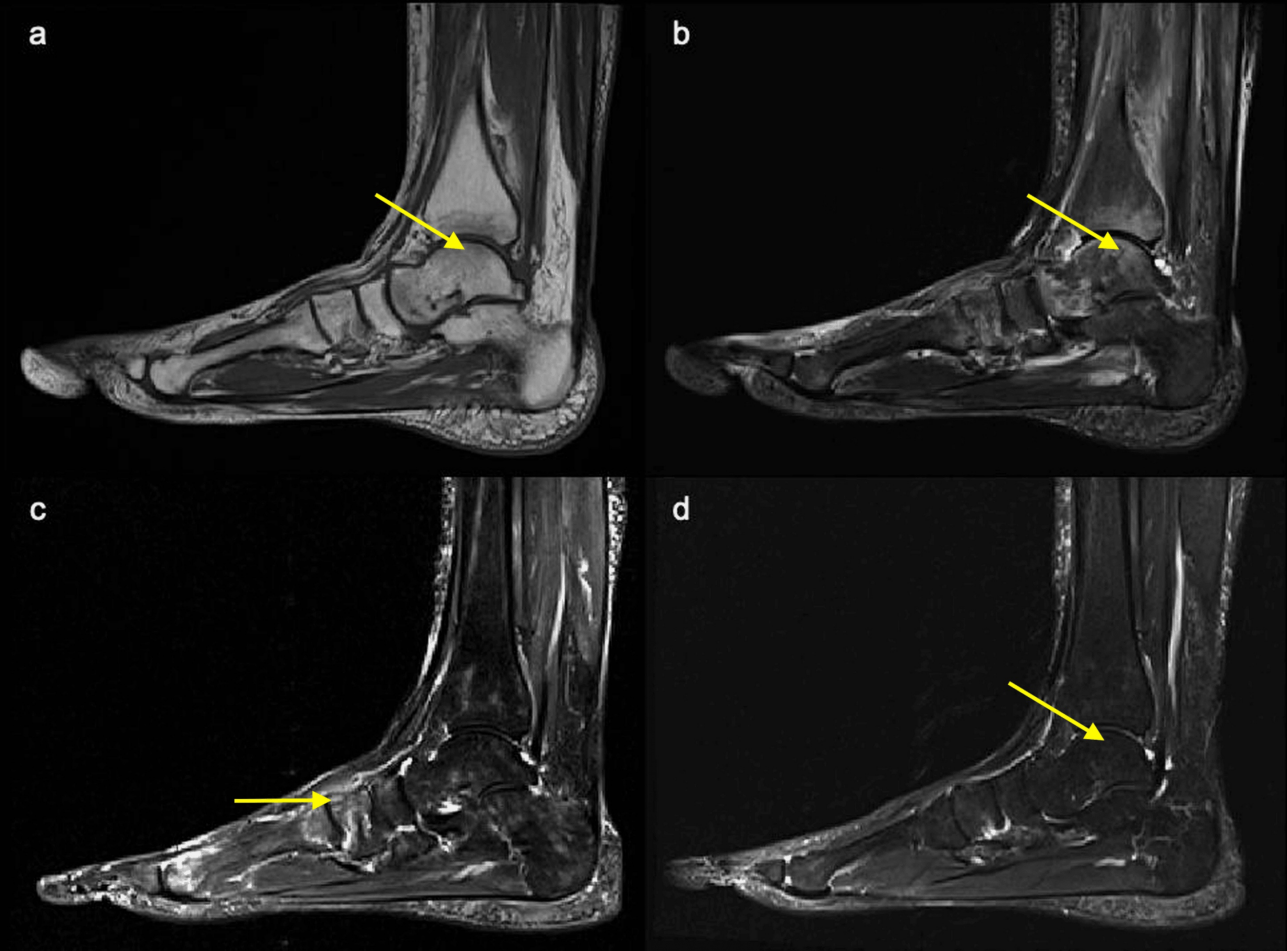
Magnetic resonance imaging (MRI) of the right ankle and foot (sagittal views). (a) T1-weighted image at one week postpartum showing low signal intensity in the distal tibia and tarsal bones. Arrow indicates a representative area in the talus. (b) Short tau inversion recovery (STIR) image at one week postpartum showing corresponding high signal intensity, indicating extensive bone marrow edema in the distal tibia, talus, calcaneus, cuboid, medial cuneiform, and proximal metatarsal bones. Arrow indicates bone marrow edema in the talus. (c) STIR image at three months postpartum showing the migration of bone marrow edema toward the forefoot. Arrow indicates bone marrow edema in the forefoot. (d) STIR image at eight months postpartum demonstrating the resolution of bone marrow edema in the ankle and foot. Arrow indicates the previously affected area in the talus.

Radiographic and clinical assessments revealed no abnormalities in other major joints, including the hips. Given that the symptoms were more pronounced in the right ankle, magnetic resonance imaging (MRI) was performed on the right side and revealed low signal intensity on T1-weighted images and high signal intensity on short tau inversion recovery (STIR) images of the distal tibia and tarsal bones (Figure 2a, 2b).

**Figure 3:**
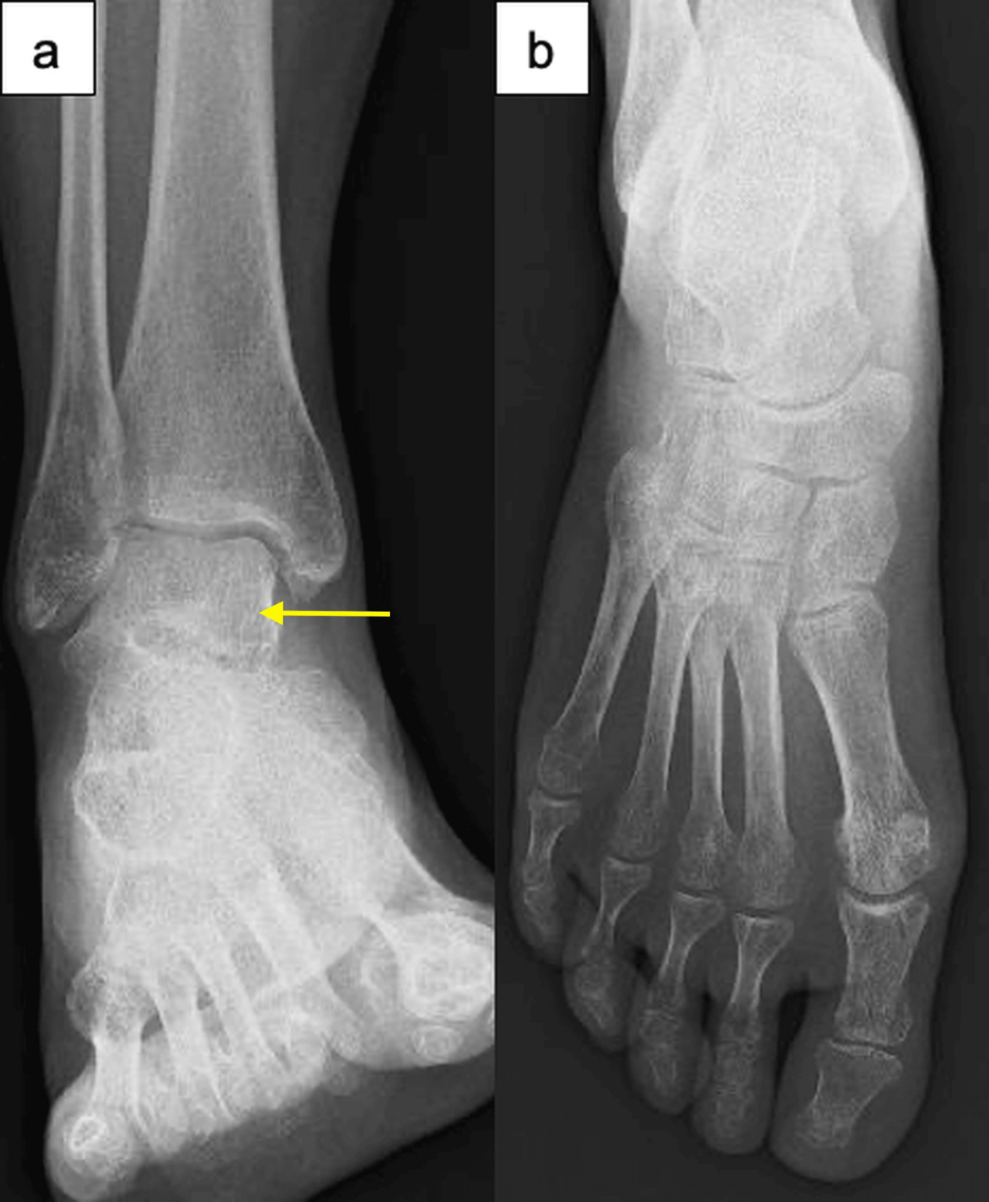
Serial anteroposterior radiographs of the right ankle and foot at 3 months postpartum. At three months postpartum: (a) partial resolution of osteopenia in the ankle; (b) improved bone density in the tarsal and metatarsal bones. Arrow indicates a representative area of improved bone density in the talus.

**Figure 4:**
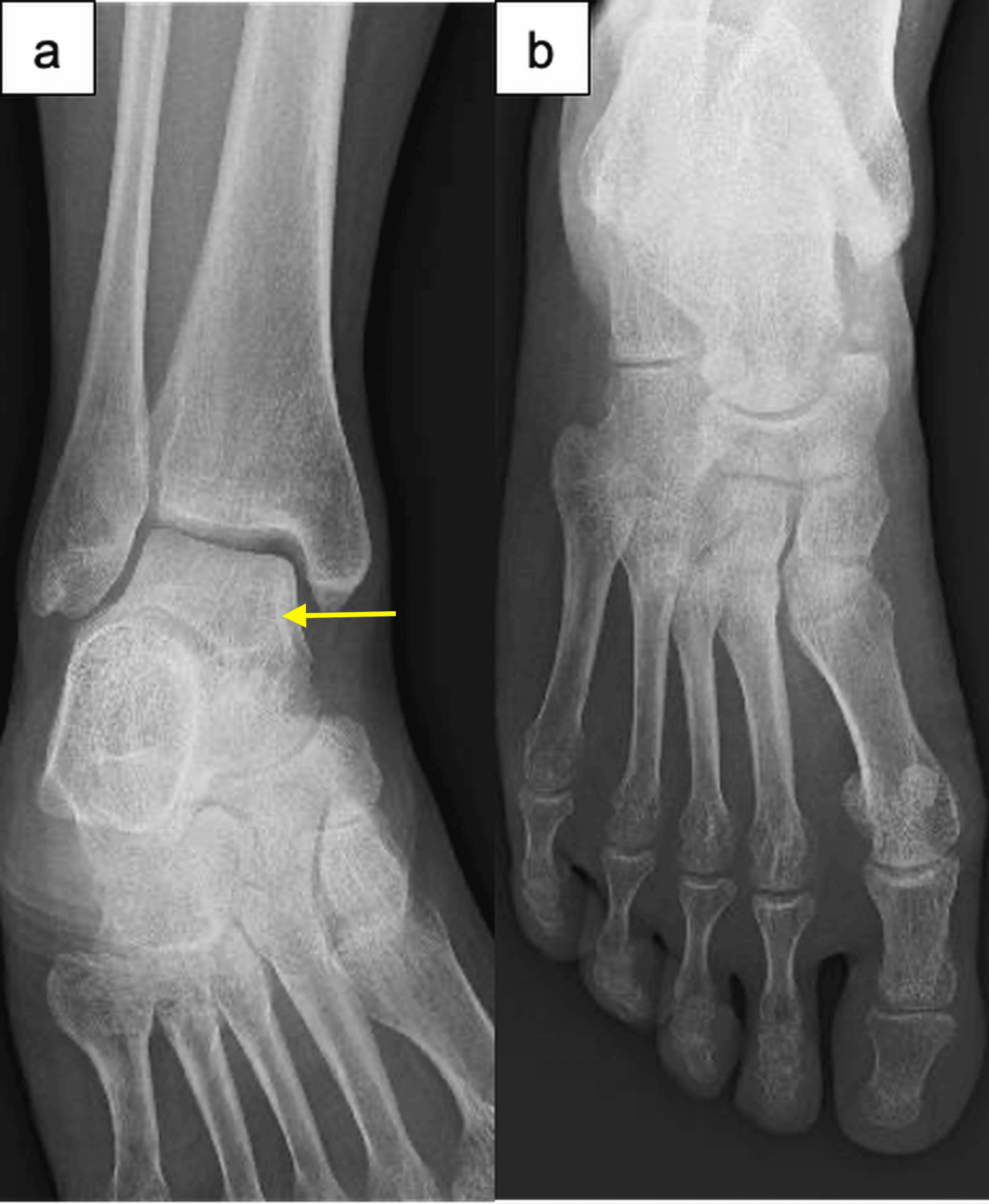
Serial anteroposterior radiographs of the right ankle and foot at eight months postpartum. At eight months postpartum: (a) complete normalization of bone density in the ankle; (b) complete normalization of bone density in the foot. The arrow indicates a representative area of normalized bone density in the talus.

These findings led to the suspicion of rheumatic diseases, and the patient was referred to the rheumatology department at the study center. She had no personal or family history of rheumatic diseases. She was an ex-smoker and did not consume alcohol. She had a height of 155 cm and a weight of 70 kg (body mass index: 29.1 kg/m2). A physical examination revealed mild swelling and tenderness on the inner side of the right ankle and mild pain in the left ankle, but no neurovascular or dermatological abnormalities. The foot arches appeared normal, with no evidence of pes planus or pes cavus. Laboratory findings for thyroid function, C-reactive protein, rheumatoid factor (RF), anti-cyclic citrullinated peptide (anti-CCP) antibodies, and antinuclear antibodies (ANAs) were normal. The levels of serum calcium, phosphorus, and bone-specific alkaline phosphatase (ALP) were also normal. However, her 25-hydroxyvitamin D level was low at 12.0 ng/mL (normal > 30 ng/mL), and she had not received vitamin D supplementation during pregnancy. Musculoskeletal ultrasound (MSUS) revealed no evidence of synovitis in the ankles. Dual-energy X-ray absorptiometry (DEXA) was not performed. The patient began a course of vitamin D supplementation, intermittent use of nonsteroidal anti-inflammatory drugs (NSAIDs), and rest with avoidance of weight-bearing on the ankles. The patient opted to continue breastfeeding. Her symptoms gradually improved during the first month postpartum and resolved completely by 3 months postpartum. At that time, radiographs demonstrated partial improvement of the patient’s osteopenia, particularly in the talus and midfoot (Figure 3a, 3b), whereas MRI revealed resolution of bone marrow edema in the tibia and talus, with new lesions appearing in the metatarsal and cuneiform bones (Figure 2c). At eight months postpartum, follow-up radiographs of the ankles revealed normalization of bone density (Figure 4a, 4b), and STIR-weighted MRI revealed no high-intensity signals in the ankles or feet (Figure 2d). The clinical and imaging findings led to the diagnosis of TOP affecting the ankles, and the patient remained free of symptoms and skeletal complications while continuing breastfeeding.

## Discussion

Our patient developed TOP affecting the ankles in the second trimester of pregnancy, earlier than the typical onset of TOP affecting the hips in the third trimester. Moreover, postpartum imaging follow-up revealed that radiographic improvement of the osteopenia preceded MRI findings, indicating the resolution, suggesting that radiographs may reflect recovery earlier than MRI and may be useful for monitoring the recovery of TOP.

Delayed diagnosis of TOP is common due to the limited use of imaging studies. Many clinicians tend to refrain from performing radiographs during pregnancy because of concerns about fetal radiation exposure. However, radiation exposure below 50 mGy is not associated with increased fetal risks, including congenital anomalies, growth restriction, and abortion [10]. Specifically, peripheral limb radiographs expose the fetus to less than 0.01 mGy [11], and hip radiographs typically range from 0.1 to 1 mGy [12]. The overestimation of radiation exposure risk may delay the diagnosis of TOP and increase the risk of complications such as insufficiency fractures. Furthermore, persistent and unexplained weight-bearing joint pain during pregnancy may impair the ability of pregnant women to perform ADLs and cause significant psychological distress.

Accurate diagnosis of TOP requires careful exclusion of other causes of regional osteopenia and bone marrow edema. Radiographic findings of regional osteopenia are not specific to TOP and may also be observed in patients with conditions such as complex regional pain syndrome, osteomyelitis, osteomalacia, inflammatory arthritis (e.g., rheumatoid arthritis), and the lytic phase of Paget’s disease of bone (PDB). PDB occasionally involves peripheral bones, including the ankles, and may radiographically mimic TOP. In contrast to TOP, PDB typically occurs in men over 50 years of age and is more common in individuals of Western European descent than in those of Asian descent [13,14]. PDB is extremely rare in young women and, to our knowledge, has not been reported during pregnancy in Asian populations [15]. Moreover, PDB typically follows a chronic course and is associated with elevated biomarkers such as ALP, which were absent in our patient. The spontaneous resolution of symptoms also supported the diagnosis of TOP over PDB.

Appropriate imaging and laboratory testing are essential for confirming TOP. In this case, DEXA was not performed, as the diagnosis was established based on characteristic clinical and imaging findings, and DEXA is not routinely required for the diagnosis of TOP. Furthermore, DEXA may not adequately reflect localized transient changes in bone density. Radiographic findings of osteopenia usually appear within 4 to 8 weeks of TOP onset. If persistent limb pain exists despite normal radiographs and TOP is still suspected, MRI should be performed to detect bone marrow edema, which is a cardinal feature of TOP. This edema can appear within 48 hours of onset and typically resolves within six to eight months postpartum [16]. While fetal harm from MRI has not been reported, gadolinium-enhanced MRI, which is usually used for evaluating synovitis, should be avoided during pregnancy due to limited safety data [10]. Serological tests (RF, anti-CCP antibodies, ANAs) and MSUS play important roles in ruling out systemic inflammatory arthritis. Although MSUS is commonly used in rheumatology, previous reports of TOP have rarely described its use. MSUS confirmed the absence of synovitis or structural abnormalities involving tendons and ligaments, serving as a noninvasive, radiation-free, and useful diagnostic tool in the differential diagnosis of TOP.

Ankle involvement in TOP remains extremely rare, with a limited number of cases reported in the literature. All previously reported cases were associated with joint pain. However, it remains unclear whether similar radiographic findings may be present in asymptomatic pregnant women. We conducted a literature review using PubMed and Google Scholar, searching for English-language articles published up to March 2024. The search terms included "transient osteoporosis" AND ("ankle" OR "foot" OR "talus") AND "pregnancy". Reports were included if they described clinical cases of TOP involving the ankle and contained sufficient imaging and clinical information.

Table 1 summarizes eight previously reported cases of TOP involving the ankles, along with the present case [4,8,17-22]. The median age at the onset of TOP involving the ankles was 33 years (range, 23-37 years). The reported cases included both primigravida and multiparous women, suggesting that parity may not be a consistent risk factor. Among the nine patients, five had bilateral ankle involvement, and six presented with symptoms in other joints, including the hips, knees, or feet. Although TOP most commonly presents in the hip during the third trimester, 5 of the 9 patients had ankle involvement beginning during the second trimester, either as the initial presentation or as part of a multi-joint onset. This earlier onset may reflect a greater percentage of trabecular bone in the tarsal bones than in the femur because bone resorption occurs preferentially in trabecular bone [23].

**Table 1:** Summary of reported cases of transient osteoporosis of pregnancy (TOP) involving the ankle Abbreviations: CT, computed tomography; Cesarean section, C-section; HELLP syndrome, hemolysis, elevated liver enzymes, and low platelet count; MRI, magnetic resonance imaging; NSAIDs, nonsteroidal anti-inflammatory drugs; CTG, cardiotocography; wks, weeks; mos, months. A hyphen (-) indicates that data were not available. *Age at first pregnancy. The same patient developed bilateral hip TOP during the second pregnancy at the age of 30, as reported in the same article.

Author	Age (years)	Affected site	Symptom onset	Diagnostic modality	Treatment	Complication(s)	Time to resolution
Ma FY et al., 2006 [17]	37	Bilateral hips, knees, and ankles	Hips: 32 wks of gestation. Knees: 3 wks postpartum. Ankles: 3 wks postpartum.	Radiograph (postpartum)	Weight‑bearing protection, NSAIDs, paracetamol	C‑section due to severe hip abduction pain	12 months postpartum
Grey A et al., 2009 [18]	33	Left ankle	20 wks gestation	Radiograph and MRI (during pregnancy), CT (postpartum)	Supportive and symptomatic treatment	C‑section due to HELLP syndrome	8 months after symptom onset
Daniel RS et al., 2009 [19]	33	Bilateral ankles	27 wks gestation	MRI (during pregnancy)	Analgesics	-	Several wks postpartum
Shenker NG et al., 2010 [4]	37	Bilateral hips, knee, left ankle	Hip: 33 wks gestation. Knee: two months postpartum. Ankle: 26 wks gestation.	MRI (during pregnancy and postpartum), radiograph (postpartum)	Pamidronate, calcium, vitamin D, analgesics	C‑section; talar insufficiency fracture	15 months after symptom onset
Uzun M et al., 2013 [8]	27	Bilateral hips, ankles	Hips: one month postpartum. Ankles: four months postpartum.	MRI (during pregnancy and postpartum)	Bed rest, paracetamol, core decompression, iloprost infusion	Sacral insufficiency fracture	17 months after symptom onset
Carranco‑Medina et al., 2016 [20]	36	Right knee, right ankle	25 wks gestation	MRI (during pregnancy), radiograph and bone scintigraphy (postpartum)	Zoledronic acid, calcium, vitamin D, analgesics	C‑section due to inability to walk	8 months postpartum
Sachsanidis P et al., 2017 [21]	25	Bilateral ankles, right foot	32 wks gestation (first pregnancy)	MRI (during pregnancy)	Calcium, vitamin D, analgesics, physiotherapy	C‑section due to abnormal CTG	9 months postpartum
Younis AA, 2021 [22]	23	Right ankle	7 months gestation	Radiograph and MRI (postpartum)	Risedronate, prednisolone, physiotherapy	-	3 months after symptom onset
Present case, 2023	31	Bilateral ankles	24 wks gestation	Radiograph and MRI (postpartum)	NSAIDs	None	3 months postpartum

Six patients underwent imaging during pregnancy: all patients had MRI scans, and one had radiographs. The remaining three patients, including our patient, were evaluated by imaging only after delivery. A total of five patients with TOP underwent cesarean section. One patient, who had TOP affecting both the ankle and knee, experienced severe functional impairment [20]. Another patient developed TOP affecting the ankles and feet during her first pregnancy and underwent cesarean section due to abnormal cardiotocography (CTG) after induction. Notably, she experienced recurrent TOP involving the hips during a subsequent pregnancy, as reported in the same article [21]. Two other patients underwent cesarean sections due to either TOP involving the hip or HELLP syndrome [17,18]. In one case [4], the indication for cesarean section was not clearly stated but was likely related to immobility from TOP involving multiple sites. This patient also developed an insufficiency fracture of the left talar dome at two months postpartum. In TOP involving the hip, non-weight-bearing or partial weight-bearing is a standard preventive strategy to avoid femoral neck fractures [24]. Given the role of the ankle as a weight-bearing joint, similar offloading approaches should be considered for patients with TOP involving the ankles. Notably, only our patient underwent serial postpartum imaging with both MRI and radiographs. At three months postpartum, radiographs revealed partial improvement of the patient’s osteopenia, whereas MRI still revealed bone marrow edema extending into the forefoot. At eight months postpartum, both had normalized. These findings may suggest that radiographs demonstrate the healing process in patients with TOP earlier than MRI does, indicating that radiographs may be a useful imaging modality for monitoring TOP.

## Conclusions

Persistent, unexplained ankle pain beginning in the second trimester of pregnancy may represent an atypical manifestation of TOP. Early diagnosis using radiographs and non-contrast MRI is essential to avoid delayed diagnosis, which may lead to functional impairment or fractures. Given the increased risk of fracture, timely initiation of conservative management is crucial. Clinicians should maintain a high index of suspicion for this condition in pregnant or postpartum patients presenting with persistent ankle pain, even in the absence of typical hip involvement. In such cases, referral to orthopedic or rheumatology specialists may be considered to facilitate appropriate imaging, diagnosis, and management.
